# Comparative Evaluation of Wettability and Contact Angle Between Endodontic Sealers and Root Canal Lumen After Using Different Irrigants: A Goniometric Study

**DOI:** 10.7759/cureus.113880

**Published:** 2026-08-03

**Authors:** Sreehitha R Reddy, Sunil Kumar C, Vamsee Krishna N, S. Sunil Kumar, K.S. Chandra Babu, Ramisetty Bharathisuma

**Affiliations:** 1 Conservative Dentistry and Endodontics, CKS Theja Institute of Dental Sciences & Research, Tirupati, IND

**Keywords:** ah plus, contact angle, endodontic sealer, goniometer, root canal irrigants, sodium hypochlorite, wettability

## Abstract

Introduction

The success of root canal treatment depends on effective chemomechanical preparation and the ability of the obturation materials to establish a durable seal with the root canal dentin. The physicochemical interaction between root canal irrigants and endodontic sealers influences sealer wettability, which plays an important role in dentinal adaptation and sealing ability. Contact angle measurement is a reliable method for evaluating wettability, with lower contact angle values indicating greater spreading ability and surface affinity. The present study evaluated the influence of two commonly used irrigants on the wettability of different endodontic sealers.

Aim

The aim of the study is to comparatively evaluate the wettability and contact angle of zinc oxide eugenol, calcium hydroxide-based, and epoxy resin-based (AH Plus) endodontic sealers on root canal dentin following irrigation with normal saline and 3.25% sodium hypochlorite using an optical goniometer.

Materials and methods

Thirty extracted human single-rooted permanent teeth were selected according to predefined inclusion and exclusion criteria. Following access cavity preparation and working length determination, biomechanical preparation was performed using 30 K hand files followed by Pro taper files up to F2 with either normal saline or 3.25% sodium hypochlorite as the irrigant. The specimens were divided into two main groups based on the irrigant used: Group A (Normal Saline) and Group B (3.25% Sodium Hypochlorite) (n = 30 each). Each group was further subdivided into three subgroups (n = 10): A1/B1 (Zinc Oxide-Eugenol Sealer), A2/B2 (Calcium Hydroxide-Based Sealer), and A3/B3 (AH Plus Sealer). A standardized 9-µL droplet of each freshly mixed sealer was dispensed onto the root canal lumen, and the contact angle was measured using an optical goniometer. Statistical analysis was performed using one-way analysis of variance, Tukey's post hoc test, and Student's unpaired *t*-test, with statistical significance set at *p* < 0.05.

Results

Significant differences in contact angle and wettability were observed among all experimental groups. Group A3 (AH Plus Sealer) demonstrated superior wettability following normal saline irrigation, whereas Group A2 (Calcium Hydroxide-Based Sealer) showed the poorest wettability. Following sodium hypochlorite irrigation, Group B3 (AH Plus Sealer) exhibited the highest wettability, followed by Group B1 (Zinc Oxide Eugenol Sealer), while Group B2 (Calcium Hydroxide-Based Sealer) showed minimal improvement. Sodium hypochlorite significantly enhanced the wettability of AH Plus and zinc oxide eugenol sealers compared with saline irrigation.

Conclusion

Group B3 (AH Plus Sealer) demonstrated the most favorable wettability among all evaluated groups. AH Plus showed superior surface characteristics, zinc oxide eugenol exhibited moderate wettability, and calcium hydroxide-based sealer demonstrated poor wettability. The combination of sodium hypochlorite irrigation and AH Plus sealer may improve sealer adaptation and obturation quality.

## Introduction

Successful endodontic treatment depends on effective elimination of microorganisms from the root canal system followed by three-dimensional obturation to prevent reinfection. Complete debridement of the root canal system remains challenging because of its complex anatomy, including accessory canals, lateral canals, and dentinal tubules. Therefore, thorough chemomechanical preparation and an effective apical seal are essential for long-term treatment success [1,2].

Root canal irrigants play a crucial role in canal disinfection, as mechanical instrumentation alone cannot completely eliminate microorganisms or remove organic debris from the intricate root canal system. In addition to their antimicrobial activity and tissue-dissolving properties, irrigants remove the smear layer, flush out debris, and modify the physicochemical characteristics of dentin, thereby influencing sealer adaptation during obturation [3,4].

Root canal sealers are used in conjunction with gutta-percha to fill irregularities, accessory canals, and the space between the core material and dentinal walls. An ideal sealer should exhibit adequate flow, dimensional stability, adhesion to dentin, low solubility, biocompatibility, and antimicrobial activity. Among these properties, wettability is particularly important because it determines the ability of the sealer to spread over dentin and penetrate dentinal tubules, ultimately improving the quality of the seal [5,6].

Wettability is commonly assessed by measuring the contact angle formed between a liquid droplet and a solid surface. A lower contact angle indicates greater wettability and better surface spreading, whereas a higher contact angle reflects poor wetting characteristics. Improved wettability enhances sealer adaptation, penetration into dentinal tubules, and resistance to microleakage, thereby contributing to successful root canal obturation [7,8].

Among the irrigants used in endodontics, normal saline serves as a biologically compatible flushing solution with minimal influence on dentin surface characteristics, whereas sodium hypochlorite remains the most widely used irrigant because of its potent antimicrobial activity and ability to dissolve organic tissue. Sodium hypochlorite has also been shown to modify dentin surface energy, potentially improving the wettability and adaptation of root canal sealers [9,10].

Several classes of root canal sealers are currently available, including zinc oxide eugenol-, calcium hydroxide-, resin-, bioceramic-, and silicone-based materials. Among these, AH Plus, an epoxy resin-based sealer, has demonstrated superior flow, adhesion, and sealing ability, whereas zinc oxide eugenol and calcium hydroxide-based sealers continue to be widely used because of their established clinical performance and biological properties [11,12].

Although previous studies have evaluated the properties of individual sealers and irrigants, limited evidence is available regarding the comparative influence of normal saline and sodium hypochlorite on the contact angle and wettability of zinc oxide eugenol, calcium hydroxide-based, and AH Plus sealers. Therefore, the present in vitro study aimed to evaluate and compare the effect of these two irrigation protocols on the contact angle and wettability of three commonly used root canal sealers using the sessile drop method with an optical goniometer.

## Materials and methods

Study design and sample selection

This in vitro experimental study was conducted to comparatively evaluate the wettability and contact angle of three endodontic sealers on root canal dentin following irrigation with normal saline and 3.25% sodium hypochlorite using an optical goniometer.

The sample size was determined using G*Power software (Version 3.1.9.7, Heinrich Heine University, Düsseldorf, Germany). Based on an anticipated effect size of 0.50, an alpha error probability of 0.05, and a study power of 80%, a minimum sample size of 60 root specimens was calculated to provide adequate statistical power.

Ethical considerations

The study protocol was reviewed and approved by the Institutional Ethics Committee of CKS Theja Institute of Dental Sciences and Research, Tirupati (Approval No. CKS/ENDO23-24/06). All extracted teeth were collected after obtaining informed consent from patients and were anonymized before use in the study.

Sample selection and storage

Thirty freshly extracted human permanent single-rooted teeth were selected according to predetermined inclusion and exclusion criteria. Soft tissue remnants and calculus were removed using an ultrasonic scaler. The cleaned specimens were stored in normal saline until further use to prevent dehydration.

Access cavity preparation

Standardized access cavities were prepared using a BR46 round bur mounted on a high-speed airotor handpiece. Straight-line access to the root canal system was established using a non-end-cutting bur.

Canal negotiation and working length determination

The canal orifices were identified using an endodontic explorer. A #10 stainless steel K-file (25 mm) was inserted into the canal until its tip became visible at the apical foramen to establish apical patency. The working length was determined by subtracting 1 mm from this measurement.

Based on the irrigant used during biomechanical preparation, the teeth were divided into two groups: Group A: Normal Saline (n = 15) and Group B: 3.25% Sodium Hypochlorite (n = 15)

Biomechanical preparation

Cleaning and shaping were performed using the step-back technique up to size 30 K-file, followed by preparation with Protaper hand files up to F2. Throughout instrumentation, intermittent irrigation was carried out using the respective irrigant assigned to each group to facilitate removal of pulpal tissue and dentinal debris.

Standardization of samples

Each tooth was decoronated at the cemento-enamel junction using a perforated diamond disc. The apical third of the root was resected to obtain standardized specimens measuring 10 mm in length.

Sectioning of specimens

The roots were sectioned longitudinally in a buccolingual direction using a low-speed diamond disc, producing two halves from each tooth while preserving the integrity of the root canal lumen.

Irrigant treatment

The prepared specimens were immersed in their respective irrigant for five minutes in sterile Petri dishes. Following immersion, the specimens were removed and blot-dried using absorbent paper points to eliminate excess surface moisture.

Mounting of specimens

Each specimen was mounted in silicone molds measuring 2.5 cm × 2.5 cm using cold-cure acrylic resin to ensure stability and standardized positioning during contact angle measurement.

Group allocation

Following mounting, these 60 root specimens obtained after longitudinal sectioning were randomly assigned to the six experimental groups (n = 10 per group) to ensure an equal distribution of specimens and to minimize selection bias: Group A1: Normal Saline + Zinc Oxide-Eugenol Sealer; Group A2: Normal Saline + Calcium Hydroxide-Based Sealer; Group A3: Normal Saline + AH Plus Sealer; Group B1: 3.25% Sodium Hypochlorite + Zinc Oxide-Eugenol Sealer; Group B2: 3.25% Sodium Hypochlorite + Calcium Hydroxide-Based Sealer; Group B3: 3.25% Sodium Hypochlorite + AH Plus Sealer.

Sealer application and contact angle measurement

The root canal sealers were mixed in accordance with the manufacturer’s instructions. The specimens were placed on the horizontal stage of the goniometer (Attension Theta Flex, Biolin Scientific, Sweden) and properly levelled. A standardized droplet (9µL) of the sealer was dispensed onto the root canal lumen using a micropipette. The software on the device quickly recorded the sessile drop profile using Young's Laplace equation for tangent fitting at the liquid-solid interface and calculated the contact angle. Multiple readings were obtained at various locations and averaged to ensure accuracy, and measurements were recorded within a specified time frame. All procedures were conducted in a controlled setting where lower contact angles indicated higher wettability.

Outcome measures

The primary outcome measure was the contact angle (degrees) of the three endodontic sealers after irrigation with normal saline and 3.25% sodium hypochlorite. Wettability was assessed by comparing the mean contact angle values among the six experimental groups.

Statistical analysis

The collected data were entered into Microsoft Excel and analyzed using IBM SPSS Statistics for Windows, Version 23 (Released 2015; IBM Corp., Armonk, New York, United States). Descriptive statistics, including mean and standard deviation, were calculated for each group. Intergroup comparisons were performed using one-way analysis of variance (ANOVA) followed by Tukey's post hoc test for multiple comparisons and the Shapiro-Wilk test for normality. A p-value of <0.05 was considered statistically significant.

## Results

Contact angle evaluation after irrigation with normal saline

According to the findings in Table 1, the tooth specimens sealed with the zinc oxide eugenol sealer (Group A1: Zinc Oxide Eugenol Sealer) demonstrated an intermediate contact angle following irrigation with normal saline, showing significantly different values compared with the other experimental sealers. The tooth specimens sealed with the calcium hydroxide-based sealer (Group A2: Calcium Hydroxide-Based Sealer) exhibited the highest contact angle following irrigation with normal saline, indicating significantly different surface characteristics compared with the other sealers, and the tooth specimens sealed with the AH Plus sealer (Group A3: AH Plus Sealer) demonstrated the lowest contact angle following irrigation with normal saline, with significantly lower values than the other two sealers.

**Table 1:** Descriptive Statistics of the Mean Contact Angle (°) for Different Sealers in Group A (Normal Saline Irrigation)

Sealer	Samples	Mean (°)	Standard Deviation	Minimum	Maximum
Zinc Oxide Eugenol	10	38.9	1.91	36	42
Calcium Hydroxide	10	71.4	4.86	64	79
AH Plus	10	27.6	3.47	22	34

Contact angle evaluation after irrigation with sodium hypochlorite

Following sodium hypochlorite irrigation, the zinc oxide eugenol sealer (Group B1: Zinc Oxide Eugenol Sealer)) demonstrated a reduction in the contact angle compared with the corresponding saline group as seen in Table 2. This reduction was statistically significant. The calcium hydroxide-based sealer (Group B2: Calcium Hydroxide-Based Sealer) continued to exhibit the highest contact angle following sodium hypochlorite irrigation, with no significant difference compared with the corresponding saline group. The AH Plus sealer (Group B3: AH Plus Sealer) demonstrated the lowest contact angle following sodium hypochlorite irrigation. Compared with the corresponding saline group, a significant reduction in the contact angle was observed (Table 2).

**Table 2:** Descriptive Statistics of the Mean Contact Angle (°) for Different Sealers in Group B (Sodium Hypochlorite Irrigation)

Sealer	Samples	Mean (°)	Standard Deviation	Minimum	Maximum
Zinc Oxide Eugenol	10	34.4	3.2	30	40
Calcium Hydroxide	10	70.5	5.48	61	79
AH Plus	10	25	2.31	22	29

The Shapiro-Wilk test demonstrated that the contact angle values in all experimental groups followed a normal distribution (all p > 0.05) (Table 3).

**Table 3:** Shapiro-Wilk Normality Test

Group	Sealer	W	p-value
A	Zinc Oxide-Eugenol	0.928	0.432
A	Calcium Hydroxide	0.977	0.947
A	AH Plus	0.979	0.961
B	Zinc Oxide-Eugenol	0.94	0.549
B	Calcium Hydroxide	0.974	0.929
B	AH Plus	0.946	0.625

Wettability evaluation

**Figure 1:**
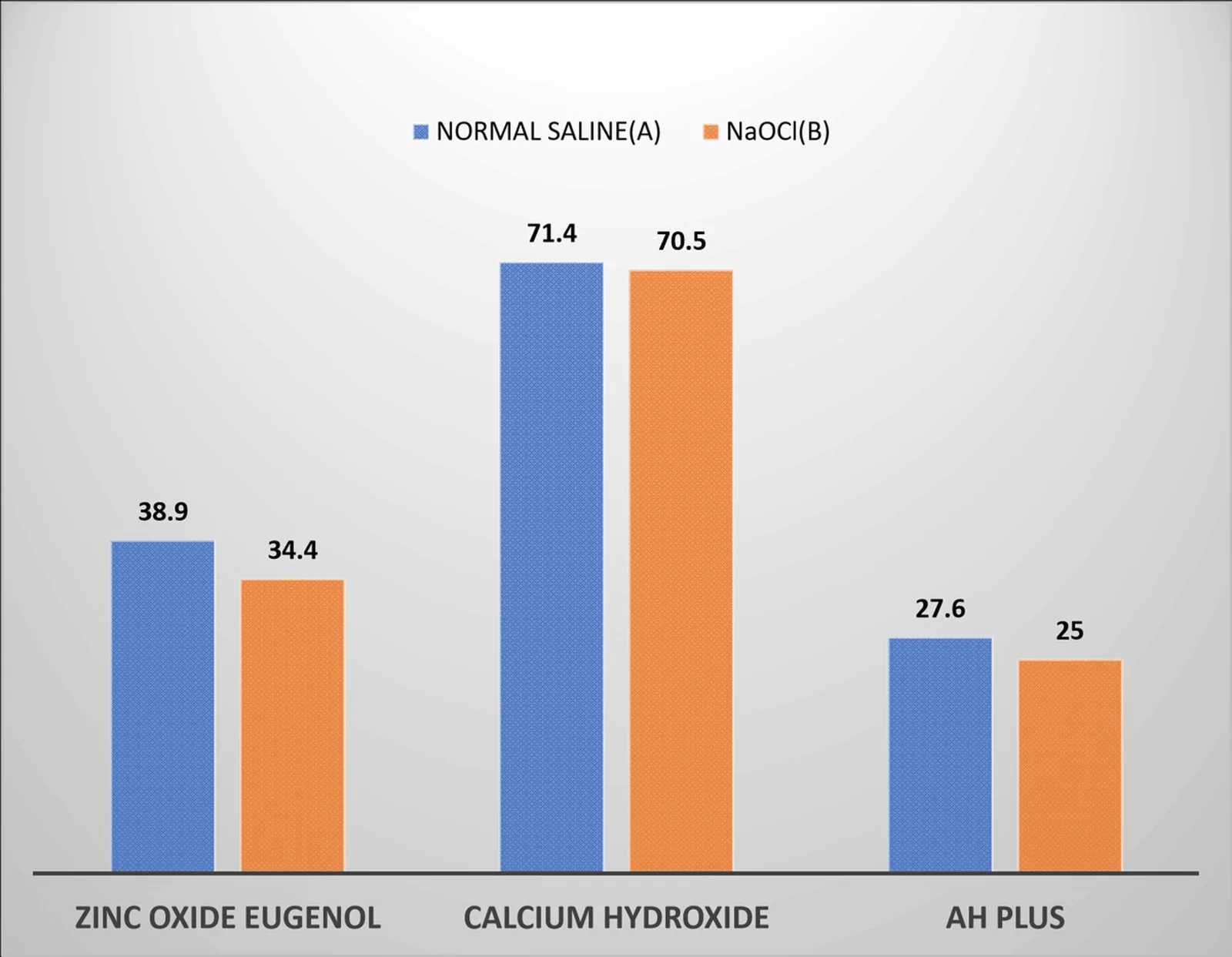
Overall Comparison of the Mean Contact Angle (°) of Root Canal Sealers Under Different Irrigation Protocols (Normal Saline vs Sodium Hypochlorite)

As seen in Figure 1, the zinc oxide eugenol sealer (Group A1: Zinc Oxide Eugenol Sealer) exhibited moderate wettability following irrigation with normal saline, demonstrating acceptable spreading characteristics on the dentin surface. The calcium hydroxide-based sealer (Group A2: Calcium Hydroxide-Based Sealer) exhibited poor wettability following normal saline irrigation, indicating limited spreading ability and surface adaptation. The AH Plus sealer (Group A3: AH Plus Sealer) demonstrated the highest wettability following normal saline irrigation, indicating superior surface spreading and adaptation. Following sodium hypochlorite irrigation, the wettability of the Zinc Oxide Eugenol sealer (Group B1: Zinc Oxide Eugenol Sealer) improved compared with the saline group, reflecting enhanced surface spreading. The calcium hydroxide-based sealer (Group B2: Calcium Hydroxide-Based Sealer) continued to demonstrate poor wettability following sodium hypochlorite irrigation, with no appreciable improvement compared with normal saline. The AH Plus sealer (Group B3: AH Plus Sealer) demonstrated the highest wettability following sodium hypochlorite irrigation, with improved spreading characteristics compared with the corresponding saline group.

## Discussion

Root canal sealers play a crucial role in achieving a fluid-tight seal by adapting intimately to the dentinal walls and filling the interface between gutta-percha and dentin. Their sealing ability is influenced by wettability, which determines the extent of sealer spreading and penetration into dentinal tubules. Irrigants not only disinfect the root canal system but also modify the dentin surface, thereby influencing the interaction between dentin and root canal sealers. Wettability is an important property that influences the ability of a sealer to spread over dentinal surfaces, penetrate irregularities, and establish close adaptation between the sealer and root canal walls. A lower contact angle indicates improved surface interaction and greater spreading ability, which may contribute to enhanced sealing performance.

 The present in vitro study was undertaken to evaluate and compare the effect of normal saline and 3.25% sodium hypochlorite on the wettability of three commonly used root canal sealers, zinc oxide eugenol, Calcium Hydroxide-based sealer, and AH Plus, using contact angle measurements.

Group A1: zinc oxide eugenol sealer with normal saline

Following normal saline irrigation, Group A1 demonstrated moderate wettability with an intermediate contact angle among the evaluated sealers. Similar findings have been reported in previous studies, where conventional zinc oxide eugenol-based sealers showed acceptable wetting ability but inferior adaptation compared with resin-based sealers [12]. The moderate wettability observed may be attributed to the material composition of zinc oxide eugenol, including its higher viscosity and limited flow characteristics, which restrict its ability to spread uniformly over dentinal surfaces. Furthermore, the absence of strong chemical interaction with dentin may reduce interfacial adaptation, resulting in comparatively higher contact angles than epoxy resin-based sealers [13].

Group A2: calcium hydroxide-based sealer with normal saline

Following normal saline irrigation, the calcium hydroxide-based sealer exhibited the highest contact angle and lowest wettability following normal saline irrigation. Previous investigations have demonstrated that calcium hydroxide-based sealers possess comparatively lower flow and increased viscosity, which negatively influences their ability to spread and adapt to dentinal surfaces [14]. The presence of particulate fillers and the absence of a resin matrix may further contribute to poor surface wetting and reduced penetration into dentinal tubules. These physicochemical limitations may explain the inferior wettability observed with this sealer in the present study [15].

Group A3: AH Plus sealer with normal saline

Following normal saline irrigation, AH Plus demonstrated the lowest contact angle and highest wettability following normal saline irrigation. This finding agrees with previous studies reporting superior wetting characteristics of epoxy resin-based sealers compared with conventional sealers [16]. The favorable wettability of AH Plus may be related to its epoxy resin composition, which provides low surface tension, excellent flow, optimal film thickness, and improved interaction with dentinal collagen. These properties enhance sealer adaptation and facilitate penetration into dentinal irregularities and tubules, contributing to improved sealing ability [17].

Group B1: zinc oxide eugenol sealer with sodium hypochlorite

Following sodium hypochlorite irrigation, the zinc oxide eugenol sealer demonstrated improved wettability compared with the saline group, as indicated by a reduction in the contact angle. Sodium hypochlorite is known to remove organic components from dentin and alter surface characteristics by increasing surface energy, thereby improving the interaction between dentin and sealer [18]. However, the wettability remained inferior compared with AH Plus, which may be due to the inherent limitations of zinc oxide eugenol, including reduced flow and limited adhesive interaction with dentin [12].

Group B2: calcium hydroxide-based sealer with sodium hypochlorite

The calcium hydroxide-based sealer showed consistently poor wettability even after sodium hypochlorite irrigation. No significant improvement was observed compared with saline irrigation, suggesting that modification of dentin surface properties by NaOCl had minimal influence on this material. The limited response may be explained by the intrinsic characteristics of calcium hydroxide-based sealers, including higher viscosity, reduced flow, and lack of resin-based bonding mechanisms. These factors may restrict spreading and penetration despite changes in dentinal surface energy.

Group B3: AH Plus sealer with sodium hypochlorite

AH Plus exhibited the highest wettability following sodium hypochlorite irrigation, demonstrating the most favorable surface interaction among all experimental groups. The improved wettability may be attributed to the combined effect of sodium hypochlorite-induced modification of dentin and the inherent physicochemical properties of AH Plus. NaOCl treatment enhances dentin surface characteristics by removing organic components and increasing surface energy, allowing better spreading and adaptation of resin-based sealers [18]. Previous studies have also demonstrated that AH Plus exhibits superior penetration, bonding ability, and sealing characteristics compared with conventional sealers.

Comparative analysis of contact angle and wettability among sealers

The overall findings demonstrated a consistent wettability pattern under both irrigation protocols, with AH Plus showing superior wettability, followed by zinc oxide eugenol and calcium hydroxide-based sealers. The lower contact angle observed with AH Plus indicates better surface spreading and potential for improved adaptation and sealing ability. In contrast, the higher contact angle associated with the calcium hydroxide-based sealer reflects poor surface interaction and limited spreading ability. Sodium hypochlorite enhanced the wettability of AH Plus and zinc oxide eugenol by modifying dentin surface properties, whereas the calcium hydroxide-based sealer showed minimal response to irrigant-induced changes. These findings emphasize that both sealer composition and irrigant selection play important roles in determining dentin wettability and the quality of root canal obturation [19].

Within the limitations of this in vitro study, the combination of sodium hypochlorite irrigation and AH Plus sealer demonstrated the most favorable wettability characteristics with the lowest contact angle compared to the other two sealers tested. Further studies incorporating long-term aging, thermocycling, and clinical conditions are required to determine whether improved wettability translates into enhanced sealing ability and long-term clinical success.

## Conclusions

Within the limitations of this in vitro study, AH Plus demonstrated superior wettability with the lowest contact angle values among the tested sealers, followed by zinc oxide eugenol and calcium hydroxide-based sealers. Sodium hypochlorite irrigation enhanced the wettability of AH Plus and zinc oxide eugenol sealers, whereas the calcium hydroxide-based sealer showed minimal change following irrigation. The findings indicate that both irrigant selection and sealer composition influence the interaction between root canal dentin and obturation materials. The combination of sodium hypochlorite irrigation and AH Plus sealer exhibited the most favorable wetting characteristics, suggesting improved potential for sealer adaptation. Further clinical investigations are required to determine whether these in vitro findings translate into enhanced long-term treatment outcomes.
